# Bone marrow mesenchymal stem cell‐derived exosomes reduce insulin resistance and obesity in mice via the PI3K/AKT signaling pathway

**DOI:** 10.1002/2211-5463.13615

**Published:** 2023-05-02

**Authors:** Hongwei Shi, Xiaojing Hao, Yaqin Sun, Huilin Zhang, Yating Zhao, Bin Wang, Jiayin Lu, Wei Hou, Yi Yan, Xiuju Yu, Linli Xue, Xiaomao Luo, Haidong Wang

**Affiliations:** ^1^ College of Veterinary Medicine Shanxi Agricultural University Jinzhong China; ^2^ Shanxi Jinnong Biological Co. LTD Jinzhong China

**Keywords:** BMSC, exosomes, inflammation, insulin resistance, obesity, PI3K/AKT

## Abstract

Obesity is a common chronic metabolic disease that induces chronic systemic inflammation in the body, eventually leading to related complications such as insulin resistance (IR), type 2 diabetes mellitus, and metabolic syndromes such as cardiovascular disease. Exosomes transfer bioactive substances to neighboring or distal cells through autosomal, paracrine, or distant secretion, regulating the gene and protein expression levels of receptor cells. In this study, we investigated the effect of mouse bone marrow mesenchymal stem cell‐derived exosomes (BMSC‐Exos) on high‐fat diet obese mice and mature 3T3‐L1 adipocyte models of IR. BMSC‐Exo treatment of obese mice promoted their metabolic homeostasis, including reduction of obesity, inhibition of M1‐type proinflammatory factor expression, and improvement of insulin sensitivity. *In vitro* analysis revealed that BMSC‐Exos improved IR and lipid droplet accumulation in mature 3T3‐L1 adipocytes treated with palmitate (PA). Mechanistically, BMSC‐Exos cause increased glucose uptake and improved IR in high‐fat chow‐fed mice and PA‐acting 3T3‐L1 adipocytes by activating the phosphoinositide 3‐kinases/protein kinase B (PI3K/AKT) signaling pathway and upregulating glucose transporter protein 4 (GLUT4) expression. This study offers a new perspective for the development of treatments for IR in obese and diabetic patients.

## Graphical abstract

Obesity is a common chronic metabolic disease that can lead to insulin resistance (IR) and type 2 diabetes mellitus‐related complications. Bone marrow MSC‐derived exosomes can alleviate obesity and IR by activating the phosphoinositide 3‐kinase/protein kinase B (PI3K/AKT) signaling pathway and upregulating glucose transport protein 4 (GLUT4) expression to increase glucose transport.

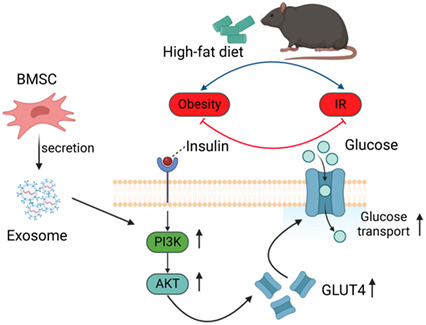

**Abbreviations**
BMSC: bone marrow mesenchymal stem cell
BMSC‐Exos: bone marrow mesenchymal stem cell‐derived exosomes
FABP4: fatty acid‐binding protein 4
FFA: free fatty acid
GLUT4: glucose transporter protein 4
GTT: glucose tolerance tests
H&E: hematoxylin and eosin
HFD: high‐fat diet
IL‐6: interleukin‐6
IR: insulin resistance
ITT: insulin tolerance tests
iWAT: inguinal white adipose tissue
MDI: adipocyte differentiation agent
NS: normal saline
NTA: nanoparticle tracking analysis
PA: palmitate
PI3K/AKT: phosphoinositide 3‐kinases/protein kinase B
scWAT: subcutaneous white adipose tissue
TEM: transmission electron microscope
TNF‐α: tumor necrosis factor‐α

Obesity has become a global epidemic. According to the World Health Organization, obesity has almost tripled since 1975, with approximately 650 million people diagnosed with obesity in 2016. The obese population is expected to increase to 12 billion by 2030 [1]. Recent data show that obesity is also strongly associated with novel coronavirus pneumonia. Obese patients are more susceptible to the virus than the general population, and obesity can exacerbate symptoms, cause poor prognosis and significantly increase mortality in patients [2]. In addition, obesity can also induce chronic systemic inflammation in the body, eventually leading to a series of related complications, such as insulin resistance (IR), type 2 diabetes and cardiovascular disease, and other metabolic syndromes [3, 4, 5]. Therefore, it is urgent to prevent the treatment of obesity and control the further development of its related complications.

Obesity refers to a state in which the body's energy intake is more significant than its energy consumption, disrupting the balance of energy metabolism in the body and causing an excess accumulation of white adipose tissue (WAT) under the skin and in the internal organs. More than 200 genes have been linked to the development of obesity [6, 7]. For example, leptin, a product encoded by the obesity gene, is produced primarily by adipocytes [8]. Leptin is overexpressed at the gene level in the adipose tissue of obese individuals [9]. Leptin, a cytokine, is elevated in circulating levels in obese patients and can lead to hypo‐inflammation [10]. Fatty acid binding protein 4 (FABP4, also known as aP2), a cytoplasmic fatty acid chaperone, is expressed mainly in adipocytes and bone marrow cells [11]. The high expression of FABP4 in the obese state of the body exacerbates many immunometabolic diseases, including diabetes and IR [12, 13]. In mouse models and humans, circulating FABP4 levels correlate with the incidence of metabolic disease, and lowering FABP4 levels or activity is associated with improved metabolic health [14].

White adipose tissue is traditionally considered the body's primary energy storage site. However, many studies have found that WAT is also a dynamic endocrine organ that secretes various cytokines, regulates communication within WAT and between WAT and other organs and cells, and participates in the body's metabolic homeostasis [15, 16, 17]. In an obese state, adipose tissue releases tumor necrosis factor‐α (TNF‐α) and interleukin‐6 (IL‐6), causing chronic inflammation in the adipose tissue, where large numbers of M1 proinflammatory macrophages are recruited to produce inflammatory factors that lead to IR in the adipose tissue [18]. In conclusion, abnormalities in the function of the adipose organs are a critical factor in the body's obesity and IR.

Insulin resistance is a condition in which the biological effect of insulin on target tissues is impaired, the efficiency of promoting glucose uptake and utilization is reduced, and the body compensates by producing excess insulin to produce hyperinsulinemia and maintain blood glucose stability [19]. Many factors can lead to IR, and obesity is one of the most important. The metabolic inflammation caused by obesity starts after WAT. Many inflammatory factors will circulate to the liver, muscle, and other insulin‐sensitive organs, interfering with the PI3K/AKT insulin signaling pathway conduction and leading to systemic IR [20, 21, 22].

With the continuous development of cell therapy technology, the quintessential role of stem cells in weight loss has been corroborated by extensive studies, and MSCs have especially attracted much attention. Studies have found that MSCs are a kind of pluripotent stem cells with proliferation, renewal, and multi‐directional differentiation, which have the functions of anti‐inflammation, immune regulation, inhibiting fibrosis, and promoting angiogenesis [23, 24]. Furthermore, it has a wide range of sources, which can be isolated from bone marrow, umbilical cord, fat, amniotic fluid, placenta, synovium, and synovial fluid [25]. Bone marrow mesenchymal stem cells (BMSCs) are mainly distributed in the femur, tibia, and iliac crest, which is easy to isolate and culture from tissues. BMSCs are considered to be one of the ideal seed cells in the field of tissue engineering since that has a strong ability for self‐renewal and genetic modification [26, 27].

It has been pointed out that MSCs repair tissue damage not only by differentiating to achieve the regeneration of damaged cells but more importantly, by releasing signaling molecules to damaged tissues through the mechanism of paracrine vesicles to enable tissue function to be restored [28, 29]. Exosomes are essential components of these paracrine vesicles and are important carriers of signal communication between stem cells and target cells [30].

Exosomes are extracellular vesicles 40–150 nm in diameter, and their contents include components such as nucleic acids, proteins, and enzymes. Exosomes are secreted by a variety of cells throughout the body, and the expression of internal components of exosomes varies depending on the source cell type and the environment in which they are located [31, 32, 33, 34]. At present, it is believed that exosomes secreted into extracellular space are recognized by target cells through ligand‐receptor binding, subsequently enter the cells through endocytosis, releasing endogenous signaling molecules to complete information transmission, and ultimately regulate target cell functions, such as promoting tissue repair and immune regulation [35, 36, 37]. The therapeutic effects of exosomes have been demonstrated in most tissues and organs. For instance, exosomes can render it possible to improve myocardial ischemia–reperfusion injury [38], promote angiogenesis to prevent diabetic nephropathy [39], promote nerve cell repair [40], and so on. The project will focus on the ameliorative effects of BMSC‐Exos on obesity‐induced inflammation and IR, providing new ideas and targets for preventing and treating obesity, IR, and related metabolic syndromes.

## Materials and methods

### Experimental animals and sample collection

C57BL/6 mice (7 weeks old, male) were purchased from the Experimental Animal Center of Shanxi Provincial People's Hospital and were maintained under constant conditions (temperature, 22 ± 3 °C; humidity, 40–50%). After 1 week of acclimatization, mice were divided into a group given a normal diet (NCD), a group of mice fed a high‐fat diet (HFD) of 60% of total calories (HDF); and a group of mice fed a HFD of 60% of total calories administered BMSC‐Exos treatment (HDF + Exosome). Mouse chow was purchased from Jiangsu Xietong Pharmaceutical Bio‐engineering (Jiangsu, China). The HDF and HDF + exosome groups were fed a HFD for 12 weeks to induce obesity. During the last 4 weeks of HFD feeding, the HDF + exosome group was treated with BMSC‐Exos, administered with an intraperitoneal injection of 50 μg of BMSC‐Exos every 3 days per animal for a total of 4 weeks. HFD or NCD mice fed with normal saline (NS) were used as controls, and their body weight and dietary intake were recorded weekly. After the intervention, mice were executed under anesthesia. Inguinal white adipose tissue (iWAT) and subcutaneous white adipose tissue (scWAT) were collected and weighed, with some tissue fixed in 4% paraformaldehyde and the rest stored at −80 °C until analysis. All animal care and experimental protocols complied with the Animal Management Rule of the Ministry of Health, People's Republic of China (Documentation No. 55, 2001) and the Guide for the Care and Use of Laboratory Animals published by the United States National Institutes of Health (Publication No. 85‐23, Revised 1996), and the Global Research Animal Guide. All animal operations were carried out in accordance with the ‘Guidelines for the Care and Use of Laboratory Animals of Shanxi Agricultural University’ and were approved by the Animal Medicine Committee of Shanxi Agricultural University [SXAU‐EAW‐2020M0725].

### Glucose tolerance and insulin tolerance tests (GTT and ITT)

For GTT, mice fasted without water for 12 h, glucose (2 g/kg body weight) was injected intraperitoneally, tail blood was taken at 0, 30, 60, 90, and 120 min, respectively, and blood glucose values were measured at different times to calculate glucose tolerance.

For ITT, mice fasted without water for 4 h and were injected insulin intraperitoneally (1 U·kg^−1^ body weight). Tail blood was taken at 0, 30, 60, 90, and 120 min, and blood glucose values were measured at different times to calculate the insulin tolerance of the mice.

### Hematoxylin and eosin

The adipose tissue was fixed with 4% paraformaldehyde for 24 h. After dehydrating and being transparent with gradient ethanol and xylene, it was embedded with wax for 4 h. Tissue sections with a thickness of 0.8 μm were prepared by an automatic rotary slicer (RM2265; Leica, Wetzlar, Germany, Japan) after embedding tissue into wax blocks. The adipose tissue sections of NCD‐NS, HDF‐NS, and HDF‐exosome groups were collected, dewaxed, and rehydrated with xylene and gradient ethanol. Tissue sections were stained with hematoxylin and eosin (H&E) and sealed with neutral glue finally.

### Cell culture

Mouse bone marrow mesenchymal stem cells and 3T3‐L1 cells were purchased from American Type Tissue Culture (ATCC) (Maryland, USA). The cells were cultured in a high glucose medium (01‐052‐1ACS; BI, Kibbutz Beit‐Haemek, Israel) containing 10% fetal bovine serum (0510; Sciencell, San Diego, California, USA) and in a constant temperature incubator (5% CO_2_, 37 °C).

### Cell treatments

After 3T3‐L1 cells were fully fused (day 1), they were induced to differentiate towards adipocytes with induction culture medium containing 0.5 mm isobutylmethylxanthine (IBMX), 0.25 μm dexamethasone (Dex), and 10 μm insulin. After 2 days of induction (day 3), cells were shifted to the insulin‐containing differentiation culture medium, and every 2 days with a change of differentiation culture, 3T3‐L1 cells were for a total of 4 days of stimulation. Mature 3T3‐L1 were adipocytes obtained for use in subsequent experiments. The inducing differentiation agents involved are referred to as MDI in the following.

Fully differentiated 3T3‐L1 adipocytes were pretreated with BMSC‐Exos (10 and 20 μg·mL^−1^) for 24 h. Subsequently, fatty acid‐free 10% bovine serum albumin medium containing 1 mm palmitate (PA) was incubated for 24 h. BMSC‐Exos continued to be administered during this procedure. PA was added to simulate the pathological condition of lipotoxicity. In addition, to investigate the insulin signaling pathway, 3T3‐L1 adipocytes were stimulated with 100 nm of insulin during the last 15 min of PA action to demonstrate the effect of PA and BMSC‐Exos on the insulin‐activated signaling pathway.

### Purification of exosomes

The BMSCs were cultured in fresh DMEM without FBS (basal medium) for 24–32 h until reaching about 85% confluency. When the number of dead cells increased under the inverted microscope, The above culture was stopped as the phenomenon appeared that the number of dead cells increased under the inverted microscope, collecting the cell's supernatant of exosome‐rich ones.

The collected cell supernatant was centrifuged at low speed at 300 ***g***, 10 min, 4 °C. Subsequently, the supernatant was centrifuged again at 2000 ***g***, 10 min, 4 °C to collect the supernatant, at which point the precipitate was dead cells and apoptotic debris. Based on the above operation, we collected the supernatant at 10 000 ***g***, 30 min, 4 °C, while the precipitate was discarded, at which point the precipitate was more giant vesicles. After centrifugation at 100 000 ***g***, 90 min, 4 °C, the supernatant was carefully aspirated to leave the precipitate washed with PBS buffer (30 mL) and resuspended before centrifugation at 100 000 ***g***, 90 min, 4 °C. The precipitate obtained after centrifugation is resuspended in 100 μL sterile PBS buffer and is ready for immediate use or storage at −80 °C.

### Transmission electron microscope

We dropped 10 μL exosome solution on copper mesh, incubated at room temperature for 10 min and rinsed with sterile distilled water, and absorbed excess liquid with absorbent paper. After absorbing 10 μL drops of 2% uranyl acetate on the copper mesh for 1 min of negative staining, the floating solution was blotted off with filter paper aiming at better results of incandescent drying for 2 min. Finally, the copper mesh was observed under a transmission electron microscope (TEM), generally with 80 kV imaging.

### Nanoparticle tracking analysis

The scattered light of nanoparticles in nanoparticle suspensions was detected after laser irradiation. The concentration of nanoparticles and their size and mass were calculated by counting the number of scattered particles as well as analyzing the particle trajectory of the exosomes.

### PKH67

With PKH67 dye (Sigma; PKH67GL, GER, Saint Louis, Germany), BMSC‐Exos were labeled, which were subsequently added to 3T3‐L1 cells for 24 h, then cells were fixed with 4% paraformaldehyde for 30 min, sealed with anti‐fluorescence attenuated blocking slices containing DAPI (S2110; Solarbio, Beijing, China), and observed with a confocal microscope (FV1000; Olympus, Tokyo, Japan). PKH67 staining was performed by utilizing a standard protocol to see the standard procedure for details.

### Cell CCK‐8

The cells containing 100 μL of the total system were seeded into the 96‐well plate (701001; NEST, Wuxi, JIangsu, China). After 12 h of preculture, differentiated 3T3‐L1 cells were treated with different concentrations of BMSC‐Exos for 24 h, and 10 μL CCK‐8 solution (40203ES80; YEASE, Shanghai, China) was added to per well at that time. The 450 nm absorbance was read using a microplate reader (filter maxF5; Molecular Devices, Sunnyvale, Silicon Valley Center, USA) after another 2 h of incubation.

### Oil Red O staining

According to the manufacturer's instructions, the Adipogenesis Assay Kit Cell‐Based (ab133102; Abcam, Cambridge, UK) was used to analyze the contents of each group's lipid droplets. Briefly, the cells were washed twice with washing solution before adding lipid droplet analysis Oil Red O solution to the cells, and the staining was observed microscopically after incubating the cells for 20 min at room temperature, after which the stained lipid droplets were detected by reading the absorbance at 490 nm with an enzymatic standard.

### Western blotting

Extracted protein samples were measured for concentration, and loadings were calculated using BCA (P0011; Beyotime, Shanghai, China). SDS/PAGE electrophoresis was performed, and the target proteins' gels were transferred to PVDF membranes (ISEQ00010; Millipore, Boston, American Massachusetts, USA). The gels were closed with 5% skimmed milk powder (abs9175; Absin, Shanghai, China) blocking solution for 2 h and incubated overnight at 4 °C. Primary antibodies include: anti‐Akt (1 : 1000, #40569, Rabbit; SAB, College Park, Maryland, USA), Phospho‐AKT (Ser473) (1 : 5000; 66444‐1‐Ig, Mouse; Proteintech, Wuhan, China), anti‐PI3K (1 : 1000, T40064, Rabbit; Abmart, Shanghai, China), Phospho‐PI3K (1 : 1000, T40065, Rabbit; Abmart), anti‐Leptin (1 : 1000, PA6011, Rabbit; Abmart), anti‐FABP4 (1 : 5000, 12802‐1‐AP, Rabbit; Proteintech), anti‐GLUT4 (1 : 5000, 66848‐1‐lg, Mouse; Proteintech), anti‐α‐tubulin (1 : 2000, 11224‐1‐AP, Rabbit; Proteintech), anti‐CD9 (1 : 1000, 20597‐1‐AP, Rabbit; Proteintech), and anti‐TSG101 (1 : 2000, 28283‐1‐AP, Rabbit; Proteintech). The target bands were incubated with HRP‐conjugated anti‐rabbit IgG (1 : 20 000, CW0156S; CWBIO, Beijing, China) for 1 h and then washed six times with TBST for 5 min each. The ECL luminescent solution detected target bands (CW0049S; CWBIO). The density of the target protein bands was normalized according to the density of α‐tubulin protein in the same sample.

### Real‐time fluorescence quantitative PCR

Primer sequences were designed using Primer Bank, and primer synthesis was performed by Shanghai General Biological Company (Shanghai, China). RNA was extracted using RNAiso Plus (9109; Takara, Japan), and the quality was tested by 1% agarose gel electrophoresis. cDNA was synthesized according to the instructions of the reverse transcription kit (R223‐01; Vazyme, Shanghai, China). SYBR (Q711‐02; Vazyme), DEPC water, cDNA, and upstream and downstream primers were mixed proportionally into a 10 μL system for a polymerase chain reaction. All samples were processed on the real‐time step one software system in triplicate (ABI QuantStudio5, Thermo Fisher Scientific, Massachusetts, USA). Results were calculated from ΔΔ*C*_T_ values. The primer sequences for qRT‐PCR used were: 5′‐TTGCTGACAGGATGCAGAAG‐3′ and 5′‐ACATCTGCTGGAAGGTGGAC‐3′ for β‐actin, 5′‐TCAAGCAGTGCCTATCCAGAAAGTC‐3′ and 5′‐GGGTGAAGCCCAGGAATGAAGTC‐3′ for Leptin, 5′‐AAGGTGAAGAGCATCATAACCCT‐3′ and 5′‐TCACGCCTTTCATAACACATTCC‐3′ for FABP4, 5′‐CACTTCACAAGTCGGAGGCT‐3′ and 5′‐CTGCAAGTGCATCATCGTTGT‐3′ for IL‐6 5′‐CCTGTAGCCCACGTCGTAG‐3′ and 5′‐GGGAGTAGACAAGGTACAACCC‐3′ for TNF‐α.

### Statistical analysis

Experiment results were presented as mean ± standard error of the mean and analyzed with one‐way analysis of variance via graphpad prism 8.3 software (GraphPad Software, San Diego, USA). Differences between the treatment group and the normal group were conducted using Student's *t*‐test. image leb (Bio‐Rad, Hercules, California, USA) was used to analyze the results of western blot analysis. The relative expression level of the target protein was calculated from the ratio of the target to the internal reference. qRT‐PCR results were calculated based on the ΔΔ*C*_T_ value.

## Result

### Identification of BMSC‐Exos

**Fig. 1:**
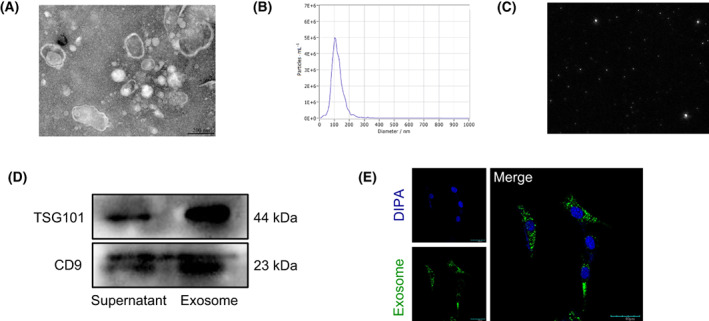
Characteristics of BMSC‐Exos. (A) The morphology of BMSC‐Exos showed a bilayer spherical vesicle structure mounted by TEM (bars = 200 nm). (B) NTA results showed that the average particle size of BMSC‐Exos was 109.4 nm. (C) Screenshot of Brownian motion video of BMSC‐Exos. (D) Western blotting detected the protein expressions of TSG101 and CD9 in BMSC‐Exos. (E) PKH67‐labeled BMSC‐Exos (green) was taken up by 3T3‐L1 cells (bars = 40 μm).

Following the isolation of exosomes from BMSC supernatant cultures by ultracentrifugation, we did observe a large number of vesicles with intact membrane structure by transmission electron microscopy, which met the internationally certified criteria for the characteristics of exosomes, showing a round or oval shape with approximately 40–150 nm in diameter, a lightly stained center and clear edges of the vesicles, and low electron‐density material was seen in the lumen of the vesicles (Fig. 1A). Nanoparticle tracking analysis (NTA) showed an average particle size of 109.4 nm and a concentration of 7.1 × 10^9^ Particles·mL^−1^ (Fig. 1B) and clearly presented the Brownian motion of BMSC‐Exos in solution (Fig. 1C). By performing western blotting, we observed positive expressions of the exosomes surface markers CD9 and TSG101 (Fig. 1D). The above results show that exosomes were successfully extracted from the supernatant of BMSC cells. To delve into the properties of exosomes, we labeled BMSC‐Exos with a PKH67 kit and added them to 3T3‐L1 cells at a concentration of 10 μg·mL^−1^ for 12 h. Subsequently, the nuclei were fixed and stained, and exosomes with green fluorescence were clearly presented to be taken up by 3T3‐L1 under confocal microscopy (Fig. 1E).

### BMSC‐Exos alleviates obesity, metabolic disorders, and inflammation in HDF‐fed mice

**Fig. 2:**
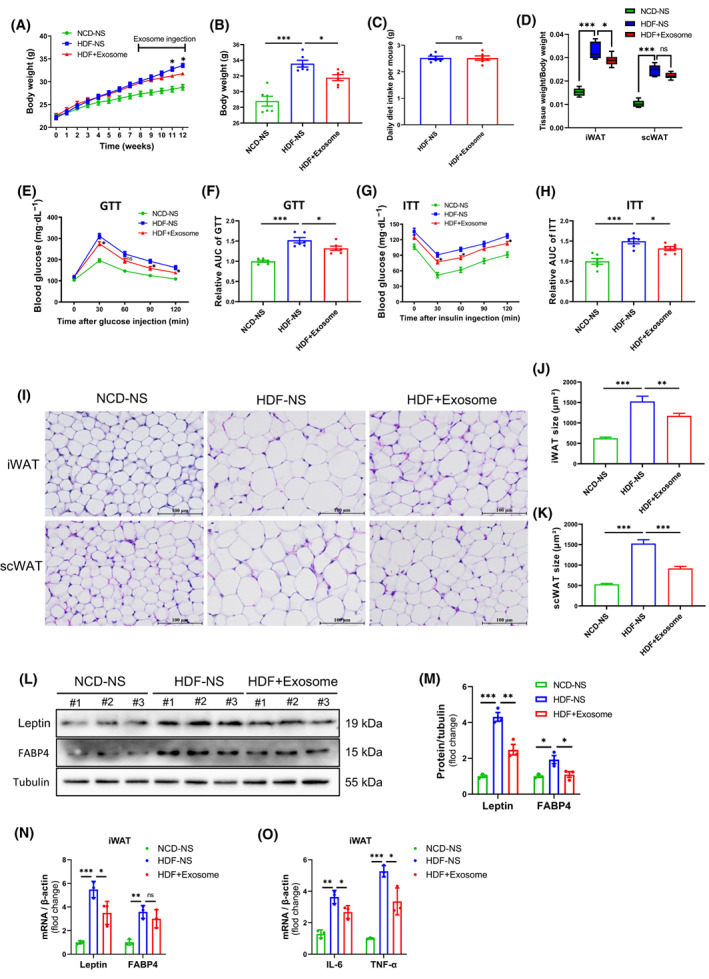
BMSC‐Exos alleviates obesity, metabolic disorders, and inflammation in HDF‐fed mice. (A–O) C57BL/6 mice were given either NCD or HDF for 12 weeks. Mice with HDF were given 50 μg of BMSC‐Exos by intraperitoneal injection every 3 days in the last 4 weeks. Equal amounts of NS were administered intraperitoneally to NCD and HDF mice as control. (A) Body weight change (*n* = 6 per group). (B) Last count of body weight of each group of mice before sampling (*n* = 6 per group). (C) Mean daily food intake per mouse in the HDF‐NS and HDF‐exosome groups during the injection of BMSC‐Exos exosomes. (D) Weight of iWAT and scWAT as a percentage of body weight for each group of mice (*n* = 6 per group). (E, F) Glucose tolerance test (GTT) and statistics of the relative area under the curve (*n* = 6 per group). (G, H) Insulin tolerance test (ITT) and statistics of the relative area under the curve (*n* = 6 per group). (I–K) iWAT and scWAT H&E staining and corresponding area statistics for each group (bars = 100 μm). (L–N) Protein and mRNA levels of iWAT obesity‐related genes (Leptin and FABP4) in various groups of mice (*n* = 3 per group). (O) mRNA levels of iWAT inflammation‐related genes (IL‐6 and TNF‐α) in all groups of mice (*n* = 3 per group). The error bars indicate the SEM, whereas comparisons between two groups were performed by an unpaired Student's test, *P < 0.05; **P < 0.01; ***P < 0.001. NCD‐NS, normal diet mice injected with normal saline; HDF‐NS, high‐fat‐fed mice injected with normal saline; HDF + Exosome, high‐fat‐fed mice injected with exosome.

Throughout the experiment, we monitored the changes in the body weight of the mice in each group. The results showed that the body weight of the mice in the HFD group was significantly higher than those in the NCD group, and BMSC‐Exos mitigated the persistent weight gain in the HDF‐fed mice (Fig. 2A,B). We counted the food intake of each group of mice during the BMSC‐Exos intervention and did not find any statistical significance (Fig. 2C). One of the key factors contributing to IR is obesity. We found that HDF‐fed mice exhibited severe glucose intolerance and IR. Administration of BMSC‐Exos significantly improved glucose tolerance and insulin sensitivity in HFD‐fed mice (Fig. 2E–H). Obesity is characterized by hypertrophy and hyperplasia of adipose tissue. We analyzed iWAT and scWAT in each group of mice. We found that HDF feeding resulted in a significant increase in the weight of both types of fat as a percentage of body weight. In contrast, exosome‐treated groups decreased iWAT weight as a percentage of body weight (Fig. 2D). H&E staining showed that adipocytes in iWAT and scWAT were significantly hypertrophied in the HDF group mice compared to the NCD group mice, while continuous administration of BMSC‐Exos significantly improved the hypertrophy of adipocytes in the HDF group mice (Fig. 2I–K). Leptin and FABP4 were highly expressed in WAT as obesity genes. Leptin, FABP4 protein, and mRNA levels were significantly higher in the iWAT of obese mice in the HFD group compared to mice in the NCD group, and BMSC‐Exos suppressed the levels of Leptin, FABP4 protein, and mRNA to some extent in obese mice (Fig. 2L–N).

Obesity is a chronic state of low‐grade inflammation, usually accompanied by the accumulation of macrophages in WAT, which secrete large amounts of inflammatory factors. In the present study, the expression of IL‐6 and TNF‐α proinflammatory factors was significantly increased in the iWAT of HFD‐fed mice, while exosome treatment reduced the mRNA levels of IL‐6 and TNF‐α in obese mice to some extent (Fig. 2O). These results suggest that BMSC‐Exos is essential in alleviating obesity, metabolic disorders, and inflammation.

### BMSC‐Exos improves PA‐induced lipid droplet accumulation and obesity in mature 3T3‐L1 adipocytes

**Fig. 3:**
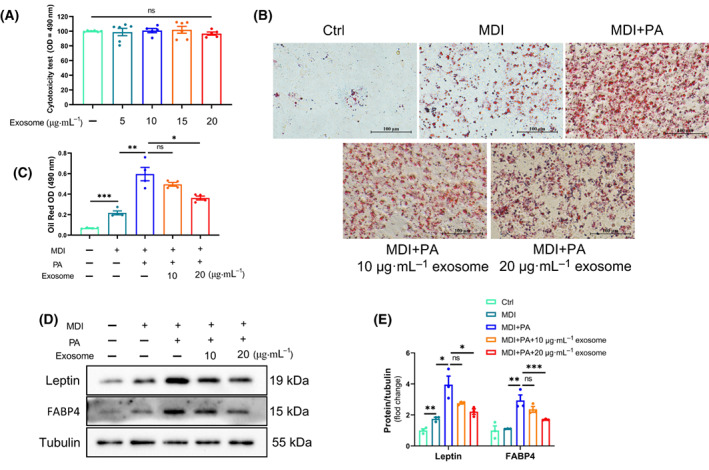
BMSC‐Exos improves PA‐induced lipid droplet accumulation and obesity in mature 3 T3‐L1 adipocytes. (A–E) 3T3‐L1 preadipocytes were added to MDI to differentiate them into mature adipocytes. Based on this, no or added BMSC‐Exos (10 and 20 μg·mL^−1^) were pretreated for 24 h, followed by adding PA to induce adipocytes in the obesity model. (A) CCK‐8 cytotoxicity assay. Different concentrations of BMSC‐Exos did not cause toxic effects on cells (*n* = 6 per group). (B, C) Analysis of lipid droplet accumulation in each group by cellular oil red O staining (*n* = 4 per group; bars = 100 μm). (D, E) Protein levels of obesity‐related genes (Leptin and FABP4) in various groups of cells (*n* = 3 per group). The error bars indicate the SEM, whereas comparisons between two groups were performed by an unpaired Student's test, *P < 0.05; **P < 0.01; ***P < 0.001. Ctrl, control group; MDI, adipocyte‐induced differentiation group; MDI + PA, adipocyte‐induced differentiation group with the addition of palmitic acid; MDI + PA + 10 μg·mL^−1^ exosome, adipocyte‐induced differentiation group treated with palmitic acid and 10 μg·mL^−1^ of exosome; MDI + PA + 20 μg·mL^−1^ exosome, adipocyte‐induced differentiation group treated with palmitic acid and 20 μg·mL^−1^ of exosome.

In order to more fully characterize the inhibitory effect of BMSC‐Exos on obesity, BMSC‐Exos was used to mature 3T3‐L1 adipocytes for relevant experiments. The different concentrations of BMSC‐Exos did not produce toxic effects on the cells (Fig. 3A). We performed Oil Red O staining to assess the effect of BMSC‐Exos on PA‐induced lipid accumulation in 3T3‐L1 adipocytes. The results showed that PA led to adipocyte hypertrophy and massive lipid accumulation, whereas BMSC‐Exos showed a dose‐dependent alleviation of adipocyte hypertrophy and lipid accumulation caused by PA (Fig. 3B,C). In addition, PA‐induced high expression of the adipocyte obesity genes Leptin and FABP4, while BMSC‐Exos also significantly and dose‐dependently reduced Leptin and FABP4 protein levels (Fig. 3D,E). These results suggest that BMSC‐Exos dose‐dependently attenuated PA‐induced.

### BMSC‐Exos regulates insulin sensitivity through the activation of the PI3K/AKT signaling pathway

**Fig. 4:**
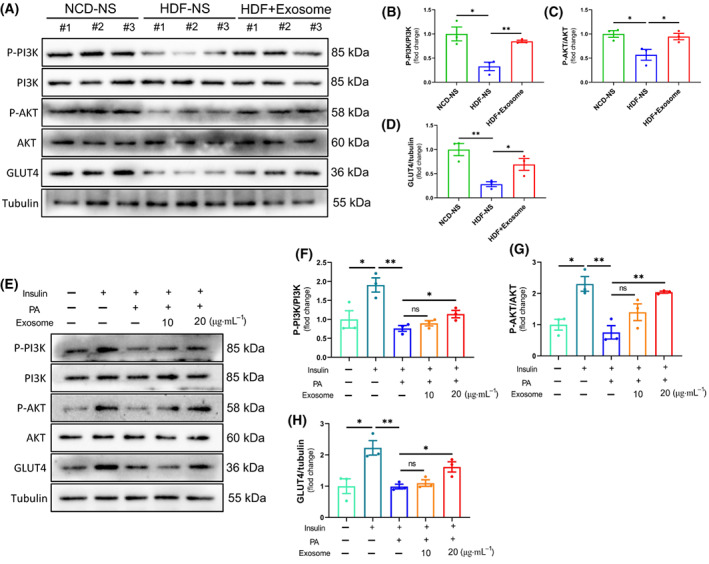
BMSC‐Exos regulates insulin sensitivity by activating the PI3K/AKT signaling pathway. (A–D) Mice were treated as described in Fig. 2, and iWAT proteins were collected for assay. Expression of each group of insulin signaling pathway‐related proteins (P‐PI3K, P‐AKT, and GLUT4) was analyzed by western blot (*n* = 3 per group). (E–H) Mature 3T3‐L1 adipocytes were pretreated with BMSC‐Exos (10 and 20 μg·mL^−1^) for 24 h. Subsequently, they were treated with 1 mm PA for 24 h and incubated with 100 nm insulin for 15 min before sample collection. Expression of each group of insulin signaling pathway‐related proteins (P‐PI3K, P‐AKT, and GLUT4) was analyzed by western blot (*n* = 3 per group). The error bars indicate the SEM, whereas comparisons between two groups were performed by an unpaired Student's test, *P < 0.05; **P < 0.01.

The PI3K/AKT signaling pathway plays a crucial role in IR. In order to further investigate the mechanism of BMSC‐Exos' role in obesity alleviation and IR, we have studied it accordingly. P‐PI3K, P‐AKT, and GLUT4 protein levels were significantly downregulated in the iWAT of HFD‐fed obese mice compared to NCD group mice, resulting in a blocked insulin signaling pathway and reduced glucose uptake and utilization in iWAT, which may lead to the pathological state of IR. Continuous administration of BMSC‐Exos to obese mice resulted in some upregulation of P‐PI3K, P‐AKT, and GLUT4 protein levels, resulting in improved insulin sensitivity (Fig. 4A–D).

To investigate the effect of BMSC‐Exos on PA interference with the insulin signaling pathway in 3T3‐L1 adipocytes, we examined P‐PI3K, P‐AKT, and GLUT4 protein levels using western blot. The results showed that the expression of P‐PI3K, P‐AKT, and GLUT4 protein levels in 3T3‐L1 adipocytes was significantly enhanced by insulin alone. In contrast, PA exposure disrupted the insulin pathway, and this effect was reversed dose‐dependently by BMSC‐Exos (Fig. 4E–H).

## Discussion

Obesity and IR are closely related and mutually reinforcing. When the body is obese, the ability of insulin to inhibit lipolysis and reduce plasma free fatty acid (FFA) concentrations is significantly impaired, leading to an increase in the rate of lipolysis and a chronic increase in plasma FFA concentrations [41]. Lipotoxicity occurs when triglycerides and their hydrolysis products, FFAs, in the blood exceed adipose tissues' metabolic and storage capacity. Large amounts of triglycerides and FFAs are transferred to non‐adipose tissues, where ectopic deposition occurs and causes tissue damage. Lipotoxicity can lead to the dysfunction of various metabolic pathways in adipose tissue and surrounding organs (liver, muscle, heart, etc.), resulting in the pancreatic cell the dysfunction and IR [42]. Increased blood lipid levels, changes in fatty acid metabolism, and alterations in intracellular signaling all contribute to IR in adipose tissue, muscle, and the liver. In this study, C57BL/6 mice were given a HFD for 12 weeks, and a mouse model of high‐fat obesity was successfully constructed, and the constructed obese mice showed signs of IR by GTT and ITT.

Exosomes are rich in quite various bioactive substances, such as nucleic acids, proteins, lipids, amino acids, and metabolites [43]. Exosomes as essential members of intercellular communication networks, embodied in their phospholipid bimolecular structure protecting their internal bioactive components from degradation or dilution to a certain extent, and also their biological functions such as transferring their bioactive substances to neighboring or distant cells through autocrine, paracrine, or telecine secretion to regulate the gene and protein expression levels of recipient cells [44]. Xu *et al*. [45], found that the pancreatic β‐cell‐derived exosome miR‐26a improved insulin sensitivity and protected β‐cell function. Wu *et al*. [46], showed that the liver‐derived exosome miR‐130a‐3p inhibits adipogenesis and thus lipid and glucose metabolism, mainly by downregulating the expression of fatty acid synthase (FASN) and PPARγ. In the present study, mouse bone marrow mesenchymal stem cell‐derived exosomes (BMSC‐Exos) were delivered to HDF‐fed mice. The results showed that BMSC‐Exos reduced body weight and iWAT accumulation and expression of obesity genes (Lpetin and FABP4) in obese mice. BMSC‐Exos also effectively alleviated systemic IR in obese mice. In addition, BMSC‐Exos was used in a PA‐induced obese 3T3‐L1 adipocyte model and effectively inhibited the accumulation of cellular lipid droplets and the expression of obesity genes.

The PI3K/AKT signaling pathway is an IR‐related signaling pathway involved in various activities, including proliferation, differentiation, regulation, and glucose transport. It is also closely associated with IR‐related type 2 diabetes [47, 48, 49, 50]. The study of the PI3K/AKT signaling pathway has helped to provide insight into the mechanisms involved in IR. Glucose metabolism depends on the cellular uptake of glucose, and GLUT is a class of carrier proteins embedded in cell membranes to transport glucose and is widely distributed in various tissues. When the PI3K/AKT signaling pathway is activated, GLUT4 is transferred from the cell to the cell membrane, increasing glucose uptake and helping to alleviate the symptoms of IR [51]. There are relevant studies demonstrating the role of exosomes in obesity‐associated IR. Yu *et al*. [52] found that adipocyte‐derived exosome miR‐27a reduced the expression of IRS‐1 and glucose transporter protein GLUT4 in skeletal muscle cells by targeting PPARγ, suggesting that adipose tissue‐derived miR‐27a may play a vital role in the development of obesity‐induced IR in skeletal muscle. Our data suggest that iWAT in HDF‐fed obese mice shows signs of IR, as observed by a significant blockage of the PI3K/AKT signaling pathway and a substantial decrease in GLUT4 protein expression. We also observed a similar PI3K/AKT signaling pathway blockage in PA‐induced mature 3 T3‐L1 adipocytes. However, when BMSC‐Exos was applied to IR models in obese mice and mature 3 T3‐L1 adipocytes, it was found that the exosomes relieved the blocked PI3K/AKT signaling pathway and inhibited GLUT4 protein expression to a certain extent, resulting in increased glucose uptake by the cells and some relief of IR symptoms.

Exosomes act as mediators to deliver content from the mother to the recipient cells, affecting human pathophysiology. Exosomes offer enormous advantages in the treatment of obesity and IR. Firstly, exosomes have low cellular immunogenicity and can avoid causing immune rejection of the organism. Secondly, exosomes contain similar content to parental cells and can act in place of parental cells. Finally, exosomes can act as a vehicle for IR by loading drugs. This project's shortcoming is that it does not provide insight into the role of specific components of BMSC‐Exos (microRNAs, proteins) in obesity‐related metabolic diseases. Nevertheless, our data suggest that exosomes derived from mouse bone marrow mesenchymal stem cells effectively alleviate obesity‐associated IR symptoms. In future, we can consider the use of exosomes to diagnose and treat obesity‐associated metabolic diseases.

## Conflict of interest

The authors declare no conflict of interest.

## Author contributions

Most of the experiments were performed by HS; HS and XH wrote the manuscript; XH revised the manuscript; YS, HZ, and YZ performed part of the *in vitro* experiments; BW and JL performed part of the *in vivo* experiments; YY, XY, WH, and LX provided technical assistance; HW and XL designed the experiments and provided funds. All authors read and approved the final manuscript.

## Data Availability

All data generated or analyzed during this study are included in this published article.
